# Cerebral small vessel disease is associated with gait disturbance among community-dwelling elderly individuals: the Taizhou imaging study

**DOI:** 10.18632/aging.102779

**Published:** 2020-02-11

**Authors:** Peixi Li, Yingzhe Wang, Yanfeng Jiang, Kexun Zhang, Qi Yang, Ziyu Yuan, Zhen Zhu, Weijun Tang, Min Fan, Weimin Ye, Qiang Dong, Li Jin, Ding Ding, Mei Cui, Xingdong Chen

**Affiliations:** 1 Department of Neurology, Huashan Hospital, Fudan University, Shanghai 200040, China; 2 State Key Laboratory of Genetic Engineering and the Collaborative Innovation Center for Genetics and Development, School of Life Sciences, Fudan University, Shanghai 200438, China; 3 Human Phenome Institute, Fudan University, Shanghai 200438, China; 4 Fudan University Taizhou Institute of Health Sciences, Taizhou 225312, Jiangsu, China; 5 Department of Epidemiology, School of Public Health, Fudan University, Shanghai 200032, China; 6 Department of Radiology, Huashan Hospital, Fudan University, Shanghai 200040, China; 7 Taixing Disease Control and Prevention Center, Taizhou 225400, Jiangsu, China; 8 Department of Medical Epidemiology and Biostatistics, Karolinska Institutet, Stockholm 17177, Sweden

**Keywords:** cerebral small vessel disease, gait analysis, walking, magnetic resonance imaging

## Abstract

Gait disturbance is considered to be a significant clinical manifestation of cerebral small vessel disease (CSVD). We aimed to investigate the association between different imaging markers of CSVD or total CSVD burden and gait disturbance in a community-dwelling population. In the cross-sectional Taizhou Imaging Study (TIS), 314 participants free of neurological disorders underwent MRI scanning and gait assessment with quantitative wearable devices as well as clinical rating scales. In linear regression, after adjustment for demographics and vascular risks, total CSVD burden was associated with prolonged 3-m walking (β=0.118, P=0.035), shorter stride length (β=-0.106, P=0.042), and poorer Timed-Up-and-Go (TUG) performance (β=0.146, P=0.009). Lacunes were positively associated with 3-m walking (β=0.118, P=0.037) and duration of TUG test (β=0.112, P=0.047). White matter hyperintensities and cerebral microbleeds were associated with prolonged stride time (β=0.134, P=0.024) and increased stance phase time percentage (β=0.115, P=0.038), respectively. Logistic regression revealed that participants with high CSVD burden or more lacunes were more likely to have an impaired gait velocity and an impaired TUG test. These results suggest that total CSVD burden and CSVD imaging markers are associated with gait disturbance among community-dwelling elderly people. Different CSVD imaging markers may cause gait disturbance through different pathways.

## INTRODUCTION

Gait disturbance is among the major causes of chronic disability in elderly populations [1] and can lead to consequences not only limited to falls, but also including loss of independence and shortening of life [2]. Although gait disturbances are prevalent in clinically normal elderly individuals, they are not merely an inevitable feature of normal ageing, but more likely to result from an underlying pathology [3]. Thus, identification of risk factors for gait disturbance is necessary for prevention and treatment.

Cerebral small vessel disease (CSVD), which is characterized by neuroimaging features including recent small subcortical infarcts, lacunes, white matter hyperintensities (WMH), perivascular spaces (PVS), cerebral microbleeds (CMBs) and brain atrophy, has been widely considered as one of the most important vascular causes of gait disturbance [4]. However, the relationship between each CSVD marker and gait performances remains inconclusive. Some previous studies have demonstrated that WMH [5–9], lacunes [8, 10] and CMBs [11] were associated with gait disturbance, but other studies found that lacunes [5, 7, 9], CMBs [5, 7, 9] and PVS [7, 9] were not associated with motor performance. Moreover, most investigations have focused primarily on general gait function by using semiquantitative clinical rating scales, chair-stand time or gait velocity for gait measurement [5, 7, 9, 10, 12], which may not be sufficiently sensitive to detect early changes in gait performance of elderly people with unrecognized CSVD. In addition, recent studies have demonstrated that the total CSVD burden might better capture the overall effect of CSVD on cognitive function [13, 14], but the relationship between total CSVD burden and gait pattern in independently living elderly people is still unknown.

The aim of our study is therefore to investigate the associations between each CSVD marker or total CSVD burden and different gait parameters by using both clinical rating scales and quantitative gait analysis in a community-dwelling population.

## RESULTS

### Demographic, imaging, and gait characteristics

The demographic, neuroimaging and gait performance characteristics of the 314 involved participants are summarized in Table 1. The mean age was 59.6 at the time of MRI acquisition and males accounted for 45.9%. Lacunes, severe WMH, CMBs and large PVS were found in 71 (22.6%), 28 (8.9%), 58 (18.5%), and 61 (19.4%) participants, respectively. The mean gait velocity was 0.95 m/s and the average time for TUG test was 10.5 s. All included subjects were divided into two groups based on total CSVD score. High CSVD burden group (with a total CSVD score ≥ 2) accounts for 17.5% of all involved subjects. No differences in age, gender and height were found between the two groups. Compared with low CSVD burden group, the high CSVD burden group had a higher proportion of current smokers, current drinkers and hypertension, although these trends were not statistically significant.

**Table 1:** Characteristics of the study population.

**Characteristics**	**Study population (n=314)**	**Low CSVD burden group (n=259)**	**High CSVD burden group (n=55)**	**Group differences (P value)**
**Demographic and clinical characteristics**				
Age, years	59.6 (2.7)	59.5 (2.8)	59.8 (2.7)	0.528
Male, n (%)	144 (45.9)	116 (44.8)	28 (50.9)	0.408
Hypertension, n (%)	166 (52.9)	132 (51.0)	34 (61.8)	0.143
Diabetes, n (%)	36 (11.5)	30 (11.6)	6 (10.9)	0.887
Hyperlipidemia, n (%)	153 (48.7)	125 (48.3)	28 (50.9)	0.721
Current smoker, n (%)	133 (42.4)	106 (40.9)	27 (49.1)	0.266
Current drinker, n (%)	99 (31.5)	76 (29.3)	23 (41.8)	0.071
Height, cm	157.2 (8.2)	157.2 (8.3)	157.1 (8.2)	0.958
**Neuroimaging characteristics**				
TIV^#^, cm^3^	1501.4 (159.5)	1499.9 (160.9)	1508.9 (153.2)	0.720
WMH volume^#^, cm^3^	1.6 (2.2)	1.1 (1.1)	3.9 (3.9)	**<0.001**
Presence of severe WMH, n (%)	28 (8.9)	0 (0.0)	28 (50.9)	**<0.001**
Presence of Lacunes, n (%)	71 (22.6)	22 (8.5)	49 (89.1)	**<0.001**
Number of lacunes	0.4 (1.0)	0.1 (0.3)	1.6 (1.9)	**<0.001**
Presence of large PVS, n (%)	61 (19.4)	38 (14.7)	23 (41.8)	**<0.001**
Presence of CMBs, n (%)	58 (18.5)	14 (5.4)	44 (80.0)	**<0.001**
**Gait performance characteristics**				
3-m walking, s	3.4 (1.1)	3.3 (0.9)	3.6 (1.7)	**0.025**
Gait velocity, m/s	0.95 (0.2)	0.96 (0.2)	0.91 (0.3)	0.136
Stride length, m	1.12 (0.2)	1.13 (0.2)	1.08 (0.2)	**0.045**
Cadence, steps/min	116.5 (11.5)	116.9 (11.4)	114.9 (11.8)	0.249
Stride time, s	1.0 (0.2)	1.0 (0.2)	1.1 (0.2)	0.387
Maximum swing velocity, m/s	3.72 (0.5)	3.75 (0.5)	3.58 (0.6)	**0.034**
Heel-strike angle, °	4.1 (6.0)	4.2 (6.0)	3.9 (6.3)	0.745
Toe-off angle, °	-59.7 (8.6)	-60.1 (8.2)	-57.6 (10.0)	**0.048**
Stance phase time, %	64.7 (2.5)	64.6 (2.4)	64.9 (2.9)	0.483
Tinetti test*	27.1 (2.1)	27.2 (1.8)	26.6 (3.1)	0.199
TUG test*, s	10.5 (3.0)	10.3 (2.5)	11.5 (4.6)	**0.009**

Data represented as mean (SD) unless other indicated. Abbreviations: CSVD, cerebral small vessel disease; TIV, total intracranial volume; WMH, white matter hyperintensities; PVS, perivascular spaces; CMBs, cerebral microbleeds; TUG, Timed-Up-and-Go.

# There are 19 (6.1%) missing data in TIV, 3 (1.0%) missing data in WMH volume.

* For skewed variables the logarithm is presented.

### Total CSVD burden and gait

**Figure 1:**
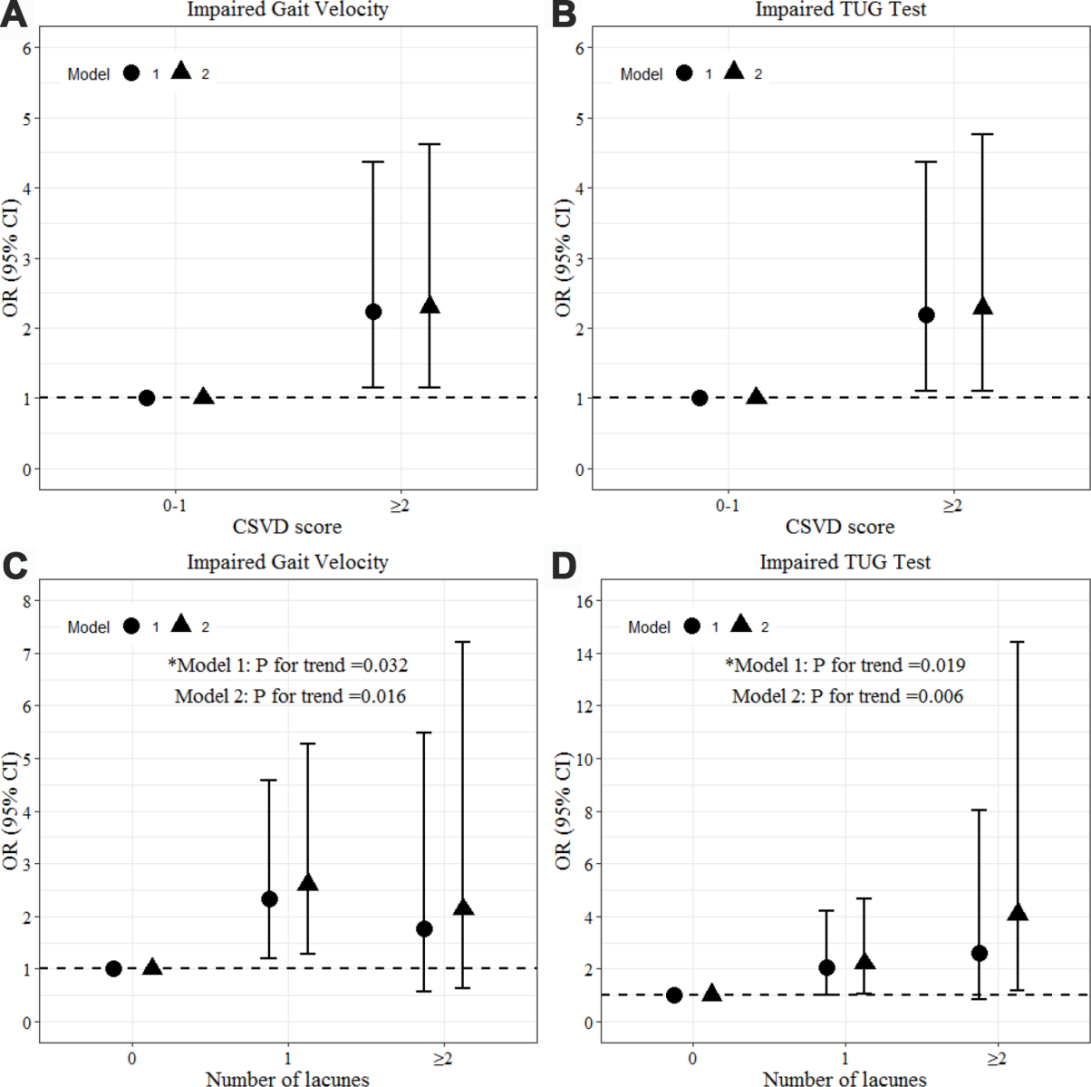
**Association between total CSVD burden, amount of lacunes and impaired gait by logistic regression.** Model 1: adjusted for age, sex and height. Model 2: additional adjustment for vascular risk factors (hypertension, diabetes, smoking, drinking, hyperlipidemia) and interval between MRI and gait measurement. Impaired gait velocity defined as < 0.8 m/s in gait velocity. Impaired TUG test defined as a TUG test of >12 seconds.

General linear models were used to assess correlations between total CSVD burden and gait performance. Total CSVD burden was grouped into high CSVD burden (defined as total CSVD score ≥ 2) and low CSVD burden. As shown in Table 2 and Table 3, after adjustment for age, sex and height, high CSVD burden was significantly associated with shorter stride length (β=-0.120, P=0.022), lower maximum swing velocity (β=-0.124, P=0.027), prolonged 3-m walking (β=0.127, P=0.025), and poorer TUG performance (β=0.154, P=0.006). After additional adjustment for vascular risk factors and time interval between MRI and gait measurement, high CSVD burden was still related to stride length, 3-m walking and duration of TUG test, and also borderline significant differences were observed in maximum swing velocity (β=-0.109, P=0.052). As gait velocity and Timed-Up-and-Go test mainly represent global gait function, logistic regression was used to identify the association between high CSVD burden and impaired gait (velocity < 0.8 m/s or TUG test > 12 seconds). Participants with high CSVD burden were 2 times more likely to have an impaired gait velocity (Figure 1A) or an impaired TUG test (Figure 1B).

**Table 2:** Association between CSVD and quantitative gait parameters.

**Outcome measures**		**High CSVD burden β (P value)**	**Lacunes β (P value)**	**WMH β (P value)**	**PVS β (P value)**	**CMBs β (P value)**
**Temporal parameters**						
Stride time	Model 1	0.049 (0.387)	0.027 (0.633)	**0.145 (0.015)**	0.039 (0.497)	0.001 (0.981)
	Model 2	0.039 (0.490)	0.024 (0.670)	**0.134 (0.024)**	0.034 (0.548)	0.001 (0.981)
Cadence	Model 1	-0.068 (0.223)	-0.044 (0.429)	-0.111 (0.059)	-0.038 (0.495)	-0.047 (0.405)
	Model 2	-0.059 (0.293)	-0.041 (0.466)	-0.098 (0.096)	-0.034 (0.548)	-0.047 (0.398)
Stance phase time percentage	Model 1	0.050 (0.366)	0.015 (0.781)	0.112 (0.053)	-0.076 (0.168)	**0.117 (0.034)**
	Model 2	0.047 (0.401)	0.010 (0.855)	0.102 (0.081)	-0.077 (0.164)	**0.115 (0.038)**
Maximum swing velocity	Model 1	**-0.124 (0.027)**	-0.078 (0.162)	-0.107 (0.069)	-0.081 (0.151)	-0.085 (0.129)
	Model 2	-0.109 (0.052)	-0.073 (0.200)	-0.095 (0.108)	-0.072 (0.196)	-0.082 (0.144)
**Spatial parameters**						
Stride length	Model 1	**-0.120 (0.022)**	-0.067 (0.200)	-0.094 (0.086)	-0.050 (0.342)	-0.083 (0.113)
	Model 2	**-0.106 (0.042)**	-0.059 (0.264)	-0.082 (0.140)	-0.042 (0.419)	-0.077 (0.143)
Heel-strike angle	Model 1	-0.037 (0.503)	0.003 (0.953)	0.017 (0.769)	-0.084 (0.127)	-0.052 (0.347)
	Model 2	-0.021 (0.711)	0.015 (0.791)	0.028 (0.630)	-0.079 (0.155)	-0.038 (0.490)
Toe-off angle	Model 1	0.107 (0.057)	0.059 (0.295)	0.088 (0.137)	0.059 (0.293)	0.011 (0.842)
	Model 2	0.102 (0.072)	0.058 (0.312)	0.088 (0.142)	0.053 (0.350)	0.013 (0.818)

β, standardized β coefficient. Model 1 represents the independent association between high CSVD burden, WMH volume in milliliters or presence of lacunes, PVS or CMBs and gait parameters adjusted for age, sex, and height, for WMH additional adjustment for total intracranial volume; Model 2 is with additional adjustment for vascular risk factors (hypertension, diabetes, smoking, drinking, hyperlipidemia) and interval between MRI and gait measurement.

**Table 3:** Association between CSVD and clinical rating scale performances.

**Outcome measures**		**High CSVD burden β (P value)**	**Lacunes β (P value)**	**WMH β (P value)**	**PVS β (P value)**	**CMBs β (P value)**
**3-m walking**	Model 1	**0.127 (0.025)**	**0.117 (0.039)**	0.099 (0.097)	0.066 (0.244)	-0.022 (0.701)
	Model 2	**0.118 (0.035)**	**0.118 (0.037)**	0.087 (0.141)	0.059 (0.291)	-0.018 (0.754)
Tinetti test*	Model 1	-0.096 (0.088)	-0.106 (0.060)	-0.023 (0.698)	**-0.112 (0.047)**	0.068 (0.231)
	Model 2	-0.083 (0.135)	-0.100 (0.076)	-0.010 (0.868)	-0.104 (0.062)	0.069 (0.218)
Timed-Up-and-Go test*	Model 1	**0.154 (0.006)**	**0.112 (0.046)**	0.112 (0.059)	0.094 (0.097)	0.050 (0.378)
	Model 2	**0.146 (0.009)**	**0.112 (0.047)**	0.102 (0.083)	0.088 (0.113)	0.055 (0.324)

β, standardized β coefficient. Model 1 represents the independent association between high CSVD burden, WMH volume in milliliters or presence of lacunes, PVS or CMBs and clinical rating scale performances adjusted for age, sex, and height, for WMH additional adjustment for total intracranial volume; Model 2 is with additional adjustment for vascular risk factors (hypertension, diabetes, smoking, drinking, hyperlipidemia) and interval between MRI and gait measurement.

*For skewed variables the logarithm is presented.

### CSVD imaging markers and gait

As total CSVD burden was shown to be related to gait disturbance, we used GLMs to assess the association between different imaging markers of CSVD and gait disturbance estimated by wearable gait tracking devices and clinical rating scales. As shown in Table 2, among quantitative temporal or spatial gait parameters, WMH volume was independently positively associated with stride time (β=0.145, P=0.015) when adjusted for age, sex, height and total intracranial volume, and further adjustments did not change the association. Presence of CMBs was associated with increased stance phase time percentage (β=0.115, P=0.038) after full adjustment. Heel-strike angle and toe-off angle were not associated with any CSVD imaging markers including lacunes, WMH, CMBs and PVS.

Regarding the clinical rating scales (Table 3), no significant associations were found between performance and the CSVD imaging markers of WMH or CMBs. Large PVS was significantly correlated with poorer Tinetti test performance after adjustment for age, sex, and height but did not survive additional adjustment. Presence of lacunes was positively associated with prolonged 3-m walking (β=0.118, P=0.037) and duration of the TUG test (β=0.112, P=0.047) after full adjustment, therefore logistic regression was further applied to evaluate the correlations between the severity of lacunes and impaired gait velocity or TUG test. As shown in Figure 1C and 1D, participants with more lacunes exhibited higher risk of impaired gait velocity (P for trend=0.016 in the fully adjusted model) and impaired TUG test (P for trend=0.006 in the fully adjusted model). For participants with 1 lacune, there was an OR of 2.6 (95%CI: 1.3 to 5.3) for impaired gait velocity and an OR of 2.2 (95%CI: 1.1 to 4.7) for impaired TUG test compared to those without lacunes after full adjustment. Those with 2 or more lacunes had a fully adjusted OR of 4.1 (95%CI: 1.2 to 14.4) for impaired TUG test and a fully adjusted OR of 2.1 (95%CI: 0.6 to 7.2) for impaired gait velocity, although this finding was not statistically significant.

## DISCUSSION

Our study assessed the relationship between CSVD and gait disturbance in an independent community-dwelling population. High CSVD burden was related to lower gait velocity and poorer TUG performance, which may be due to shorter stride length and lower maximum swing velocity. Among four CSVD imaging markers, lacunes were independently associated with impaired gait velocity and TUG test, indicating that lacunes were strongly correlated with global gait function. WMH volume and presence of CMBs were associated with prolonged stride time and an increased stance phase time percentage, respectively. These results suggest that participants with higher WMH volume or CMBs may be more likely to develop an early stage of gait impairment, in which no apparent gait disturbance, such as impaired gait velocity or TUG test, are exhibited. No associations were found between presence of large PVS and gait parameters.

The relationships between individual CSVD imaging markers and gait performance remain controversial [5, 7–11]. Our study revealed a dose-dependent effect between number of lacunes and presence of impaired TUG test. However, such relationship did not apply to gait velocity, as the odds ratio for 2 or more lacunes was not significant, which may be due to a limited sample size (n=16 for participants with ≥ 2 lacunes). One possibility for the difference is the added elements of standing, turning and seating in the TUG test, such that it may provide more information about functional mobility than gait velocity [15]. There have been conflicting results in the relationships between lacunes and gait velocity or TUG performance among previous studies. Consistent with our study, the Radboud University Nijmengen Diffusion tensor and MRI Cohort (RUN DMC) study [8] and the PURE-MIND study [10] have reported that lacunes were significantly associated with gait velocity and TUG performance, respectively. However, the Lothian Birth cohort 1936 (LBC1936) study [7] and the Shunyi study [9] found no associations between lacunes and gait velocity or TUG performance. This may be explained by a reduction in the strength of observed correlations due to the lower prevalence of lacunes in their study population compared with participants in our study (5.7% vs. 22.6%), coupled with the absence of adjustment for height [7], and the lack of evaluation of the severity of lacunes [9]. Contrary to previous large cross-sectional studies [5, 7–10], our study found that WMH was correlated with prolonged stride time, but not with gait velocity, TUG test or other parameters. This result suggested that those participants with severe WMH may spend more time to complete one gait cycle, which indicated that WMH may affect gait fluency. However, several differences between studies need to be considered. Our study population was younger in comparison to previous studies of community-dwelling subjects, which lead to a lower WMH burden in our study. Thus, participants in our study were more likely to present with subtle gait disturbances. Additionally, WMH volume was obtained in our study, which may represent the severity of WMH more accurately than the Fazekas scale [16], which is semi-quantitative and likely to be influenced by subjective factors. In consideration of cerebral microbleeds, previous population-based studies found no associations between CMBs and gait speed or chair-stand time [7, 9]. The RUN DMC study, carried out in 485 elderly patients with CSVD, found that a higher number of CMBs was associated with shorter stride length and poorer performance on the Tinetti and TUG test, but did not involve stance phase time in gait measurement [11]. In our study, a novel finding was that CMBs were significantly associated with an increased stance phase time proportion, indicating that independent elderly people with CMBs may be more likely to suffer from an unstable gait. Consistent with previous studies [7, 9], our study suggests that PVS may not be severe enough to cause a functional impact on its own.

Emerging studies have demonstrated that total CSVD score, which summarizes individual CSVD imaging markers in a compound scale, may better capture the overall effect of CSVD on functional impairment [7, 13, 14, 17–21]. However, gait disturbance has rarely been a target outcome among such studies, especially in population-based studies examining the clinical consequences of CSVD. In the current study, we found that a total CSVD score of two or above was significantly associated with several gait parameters. Accordingly, in participants with a high CSVD burden, we assumed a gait pattern with shorter stride length and lower swing velocity, which lead to slower gait speed and poorer global gait function.

The pathogenesis of gait disturbance due to CSVD has not yet been identified. Some previous studies have demonstrated that a disruption of white matter integrity [5, 6, 22] or brain atrophy [5, 23] may be responsible for gait disturbance in participants with CSVD, with different locations or patterns relating to specific gait parameters. For instance, stride length was found to be associated with the volume of frontoparietal regions and visual association cortices [23, 24], and white matter integrity in corpus callosum and corona radiata [6]. Cadence was positively correlated with the thickness of primary and supplementary motor cortices and the cingulate cortex [23]. These findings suggest that different cerebral networks are involved in various gait parameters. Accordingly, we highlighted distinguishing gait patterns of different CSVD markers in the current study, which indicated that CSVD markers may result in gait disturbances through different pathways. However, further studies with neuroimaging techniques are needed to clarify the underlying mechanisms in the association between gait disturbance and CSVD.

A strength of our study is that we measured gait function in a quantitative manner. As a previous study has reported [8], some quantitative parameters could lead to early detection of gait abnormalities instead of gait velocity, which indicated that the early alterations of these quantitative parameters can be subclinical and not enough to cause a clinically recognized impairment on their own. The use of quantitative gait tracking devices may help detect small differences in these parameters between groups, therefore providing a more sensitive method for assessing subtle gait disturbances. Importantly, we included heel-strike angle, toe-off angle and stance phase time percentage in a CSVD-related gait analysis, as these parameters have been confirmed to be able to distinguish PD patients from controls [25]. However, altered heel-strike angle or toe-off angle, which indicate shuffling gait and have been shown decreased in patients with Parkinson’s disease [25], were not detected in participants with CSVD. We also included TUG test and Tinetti test in our study as these tests are frequently used and easy to apply in everyday clinical practice, as well as having been applied in previous studies focused on gait disturbance in CSVD. Duration of TUG test was significantly related with total CSVD burden and lacunes. We therefore suggest that the TUG test could be a sensitive indicator for subclinical CSVD. However, Tinetti test seemed to be insensitive in capturing CSVD related gait disturbances during our study.

Our study has several limitations. First, musculoskeletal problems were not considered in our study, which may affect gait performances. However, all participants involved were able to walk normally. Second, although there were no clinically diagnosed neurological disorders in our participants, subclinical pathologies related to neurodegenerative diseases were not able to be excluded. Third, our study was cross-sectional and did not address causal relationship. Hence, large longitudinal cohort studies are still required to provide further evidence on the causal relationship between CSVD and gait disturbance. Fourth, some of the associations were only of borderline statistical significance in our study, which may be due to a relatively small sample size. Furthermore, participants in our study were younger compared with participants in previous community-dwelling studies and without complaints of gait abnormalities, making it harder to show significant differences. Finally, the inclusion of only rural villagers from Taizhou limits the generalizability of our results. We are currently expanding the TIS and following all of the participants longitudinally, which will help clarify the causal relationship between CSVD and gait disturbance based on a larger population.

In summary, our study confirmed that total CSVD burden and CSVD imaging markers were associated with gait disturbance among community-dwelling elderly people in a rural Han Chinese population. Different CSVD markers may cause gait disturbances through different pathways. The total CSVD score is a possible marker to identify non-disabled elderly individuals at risk of gait impairment. To confirm our findings, a longitudinal cohort study featuring a larger sample size is warranted.

## MATERIALS AND METHODS

### Study population

For the present study, we used the baseline data from Phase I of the Taizhou Imaging Study (TIS), an ongoing population-based cohort study nested within the Taizhou Longitudinal Study (TZL) [26]. TIS was designed to investigate the risk factors and consequences of structural and functional brain changes in community-dwelling elderly people in a rural Han Chinese population. In 2013, two villages with the previous highest response rate were chosen from TZL and involved in the TIS. Inclusion criteria were: 1) aged between 55 and 65 years; 2) being Han Chinese whom have settled at Taizhou for over the past 10 years and lived locally for more than 9 months per year; 3) able to walk and communicate normally and independently, and able to provide information by self-reporting and participate in physical examinations. Exclusion criteria were: 1) history of stroke or cancer; 2) history of clinically diagnosed neurological or psychiatric disorders; 3) signs of abnormalities in neurological examination; 4) prominent visual or hearing impairment. Accordingly, a total of 624 participants were involved. Among them, 562 individuals responded and agreed to participate in our study. The response rate of participation was 90.1%. Informed consent has been obtained. The Ethics Committee of the School of Life Sciences, Fudan University, Shanghai, China approved the study (institutional review board approval number: 469).

At the baseline, all of 562 participants accepted questionnaire interviews, physical and laboratory examinations and brain MRI scans, 317 of them accomplished quantitative gait analysis. From the 317 participants, we excluded 2 with incomplete data and 1 with current mobility aid use due to fracture, leaving 314 for the current analysis.

### Collection of clinical characteristics

As reported previously [27, 28], current smokers were defined as individuals that reported regular smoking for at least 6 months before enrollment; current alcohol drinkers were individuals consuming more than three drinks per week for at least 6 months before enrollment. Blood pressure was measured in the right upper limb in a sitting position after resting for 10 minutes in a quiet room. Hypertension was defined by systolic blood pressure ≥ 140 mmHg or diastolic blood pressure ≥90 mmHg, a previous diagnosis of hypertension, or use of antihypertensive drugs. Venous blood samples were collected by certified nurses between 7 AM and 8 AM after overnight fasting. Diabetes was determined by a fasting level of plasma glucose ≥ 7.0 mmol/L, a previous diagnosis of diabetes, or treatment with antidiabetic drugs. Hyperlipidemia was defined by plasma total cholesterol (TC) ≥ 5.2 mmol/L, triglycerides (TG) ≥ 1.7 mmol/L, a previous diagnosis of hyperlipidemia, or current use of lipid lowering drugs. All examinations were conducted at the Taizhou People’s Hospital, Taizhou City, Jiangsu Province, China.

### MRI scanning and processing

MRI examination for all participants were performed on the same 3.0-Tesla scanner (Magnetom Veiro Tim scanner; Simens, Erlangen, Germany) with a pre-determined multi-sequence protocol. The MRI sequences included T1−weighted, T2-weighted, fluid attenuated inversion recovery (FLAIR), T2^*^−weighted gradient recalled echo (GRE), proton density-weighted imaging (PDWI), perfusion-weighted imaging (PWI), diffusion tensor imaging (DTI), and time of flight (TOF) 3D magnetic resonance angiography (MRA). The specific parameters of each sequences have been reported previously [28, 29].

Two experienced neurologists (M.C. and Q.Y.) were involved in reading and assessment of MRI images. Each of them read images independently and the final report was based on their consensus. Conflicts during assessment were handled to a senior neuroradiologist (W.T.) for a final judgment. The inter-rater agreements were tested by Cohen’s kappa statistics and showed good reliability (kappa=0.81). Lacunes, WMH and CMBs were diagnosed based on the Standards for Reporting Vascular Changes on Neuroimaging (STRIVE) criteria proposed in 2013 [4]. Presence of severe WMH were defined as those with either periventricular white matter hyperintensities (PVWMHs) of deep white matter hyperintensities (DWMHs) rated at least two on the Fazekas scale [16]. As recent studies revealed that large PVS, compared with small PVS with a maximum diameter ≤ 2 mm, may have a stronger relevance with CSVD-related functional deficit, we defined PVS as round or tubular lesions with CSF-like signal and a short axis ≥ 3 mm in the subcortical area in the current study. Imaging markers of CSVD, including lacunes, CMBs, severe WMH and PVS, were summed in an ordinal “total CSVD score” (with a range of 0–4) by counting presence of each of these four MRI features [14].

The volume of WMH was determined using SPM8 software [30]. Total intracranial volume (TIV) was estimated using FreeSurfer v6.0.0 software (http://surfer.nmr.mgh.harvard.edu/fswiki/). The results of SPM and FreeSurfer were reviewed for accuracy by the consensus of two experienced neurologists (Y.W. and H.W.) There were 19 (6.1%) missing data in total intracranial volume (TIV) and 3 (1.0%) missing data in WMH volume due to motion artifacts and extreme outliers to maintain imaging quality control.

### Measurement of gait

Quantitative gait analysis was performed with an insole-like wearable gait tracking device (Senno gait, Sennotech Co. Ltd., China) [31]. A result of comparison testing, by the China National Institute of Standardization (CNIS) showed that this wearable gait analysis system accurately captured gait parameters as compared to a widely used optoelectronic motion capture system (Vicon, Oxford Metrics, UK). Participants walked freely at a comfortable, self-chosen speed in an obstacle free and flat environment for 2×10 meters. After the first 10-meter walk, participants were instructed to turn 180 degrees. Gait parameters were extracted from each stride and data were streamed wirelessly via Bluetooth^®^. Data were automatically ordered and the top and bottom 15% were excluded thus turning strides would not be analyzed. Temporal parameters included stride time (s, the duration of one gait cycle), cadence (the number of steps per minute), stance phase time percentage (%, the portion of the cycle during which part of the foot touches the ground) and maximum swing velocity (m/s, the maximum forward speed of the foot during swing). Spatial parameters included stride length (m, the distance between the heel points of two consecutive footprints of the same foot), heel-strike angle (°, the angle between the foot and the ground at heel strike on a vertical plane) and toe-off angle (°, the angle between the foot and the ground just at take-off on a vertical plane). Clinical rating scales assessment consisted of the Tinetti test [32] with 17 items (9 for balance and 8 for gait) with a maximum score of 28 as well as the Timed-Up-and-Go (TUG) test [33]. For measurement of gait velocity, 3-m walking test was performed. Participants walked at usual pace on a 3-m course and time for walking was measured by a stopwatch.

### Statistical analysis

Continuous variables were expressed as mean and standard deviation, and categorical variables were expressed as frequencies and proportions. Shapiro-Wilk test was used to evaluate the normality of continuous variables, and logarithmic transformation was applied for skewed variables. The two-sample t-test or chi-square test was applied to evaluate group differences according to requirement.

In consideration that participants with 2 or more CSVD imaging markers constituted only 17.5% of the study population, high CSVD burden was defined as a total CSVD score ≥ 2 in the current study. To evaluate the relationship between low/high CSVD burden and gait performances, general linear models (GLMs) were applied with the group as a determinant and quantitative gait parameters (stride time, cadence, stance phase time percentage, maximum swing velocity, stride length, heel-strike angle, toe-off angle) or clinical rating scale performances (3-m walking, Tinetti test and Timed-Up-and-Go test) as outcome variables. Covariates included age, sex, height, vascular risk factors (hypertension, diabetes, smoking, drinking, hyperlipidemia) and interval between MRI and gait measurement. The relationships between CSVD imaging markers and gait performances were investigated by using similar GLMs with each CSVD marker (WMH volume, presence of lacunes, presence of PVS and presence of CMBs) as a determinant. For analyses between WMH volume and gait, total intracranial volume was additionally adjusted. The risk of impaired gait by high CSVD burden or the severity of lacunes was assessed with logistic regression analysis with adjustment for age, sex, height, vascular risk factors, and interval between MRI and gait measurement. As a previous study reported that there were significant differences between multiple lacunes and single lacune in vascular factors and prognosis [34], the severity of lacunes was grouped as none, one and two or more lacunes in our study. According to previous studies, impaired TUG test was defined as a duration of TUG test >12 seconds [35]. Impaired gait velocity in this study was defined as a gait velocity under the first quartile (< 0.8 m/s).

Statistical analysis was performed using SPSS version 21.0. A probability value of < 0.05 was considered significant.
